# On Toxic Neutralisations

**Published:** 1894-09-29

**Authors:** Benjamin Ward Richardson


### ON TOXIC NEUTRALISATIONS.

By SIR BENJAMIN WARD RICHARDSON, M.D., F.R.S.

### I.—ISOMERIC VARIATION.

WHEN, some thirty years ago, a change of method in investigations of a therapeutical kind was taking place and experiment was receiving a strong impulse in what was then felt to be a new and exact direction a few of us were busied trying to determine whether we could neutralise the effect of one therapeutical substance in the body by the administration of another. Some of us came to the conclusion that we might do so, and I, for one, was strongly of opinion that this curious art of medicine was within our reach, always assuming that we were dealing with organic compounds, that is to say, medicinal substances that admitted of chemical modification of their composition under the influence of some other compound which seemed to possess neutralising powers. As we progressed we soon suspected that we had to confine our research to one issue. We could not neutralise the elementary or the binary forms of matter, such as phosphorus or the oxides of metals, like an arsenical oxide. Those substances were too basic, and their immediate or primary action was too destructive to be checked when once commenced. In regard to phosphorus Köhler was hopeful, but it never seemed to me that he advanced far in direction of actual discovery, and as after his early death no one of the same intelligence and originality took his place, new research was limited to the organic series, and the work implied on them alone was, after all, sufficient for considerable time and labour, if it had been as definitely successful as some of us had, perhaps rather hastily, expected. The truth is that certain serious difficulties stood in our way, which, for the benefit of future observers, I shall do well to notice.

*The Isomeric Difficulty.*—In my own researches I soon discovered the singular fact that something more than the mere elementary constitution of a medicinal or antidotal organic substance had to be taken into account. There was the little understood question of the combination of elementary forms before us for contemplation. The pure chemists were brought to a pause here in their dealing with matter comparatively steady and regular in its transmutation, dead; how much more difficult then our task who were dealing with agencies that had to come under the modifying action of the motion called, obscurely, living motion, or life? We had to meet the strange problems of isomeric differences. It turned out, that is to say that isomeric substances—substances that are identical in chemical composition—did not, in a physiological point of view, produce the same identical action on living bodies under their influence. For example, formiate of ethyl is chemically isomeric with acetate of methyl, and might be supposed, therefore, to be physiologically isomeric, if that expression be permitted. But when the two substances came to be tested in a physiological manner it was found that their effects on the living body were distinctive.

The vapour of the formiate of ethyl when inhaled produced a slight stupor but no actual sleep, while there was caused by it considerable muscular excitement and nausea with vomiting if the inhalation were carried on long enough. The vapour was also irritating to the throat and to the bronchial passages. These results were very decisive, and left no doubt as to physiological effect; the formiate of ethyl is strictly an irritant, and is strictly not a narcotic; it might be narcotic since there is about it a tendency to act as such, but its action as an irritant interferes and raises a new and opposing condition.

The action of the isomeric acetate of methyl was singularly diverse. This acetate on being introduced into the body caused a deep stupor, unattended with any sign of muscular excitement, vomiting, or other irritation. Acetate of methyl was, in fact, a narcotic, and not an irritant.

These two bodies are not apparently very complex in a chemical sense. Three elements only, viz., carbon, hydrogen, and oxygen ($C_2 H_6 O_2$) enter into their composition; yet their internal combination must be such as to make them decidedly unlike. In the formiate the element hydrogen seemed physiologically to be playing the same part as it does in ammonia, and as it does in many other compounds in which it is present, and the convulsive movements were much like those which are brought about by the inhalation of carbonic oxide, and indeed the inhalation of carbonic oxide and ammonia vapour diluted in air led to precisely the same symptoms; whilst in methyl acetate the hydrogen appeared to have lost its power as an irritant, as in several of the compounds in which it is an element, so that the effect produced was all but identical with the action of carbonic acid in combination with methylic ether.

These effects on the body of isomeric substances led me to many ideas of a novel kind respecting the change of functions under medicinal agents of such important structures and organs of the body, as the brain, spinal cord, sympathetic centres, and blood. I could not help reasoning, and cannot help it still, that without losing their elementary basis, many bodily parts, as well as therapeutical substances, may, under different environing conditions—differences of temperature, electricity, moisture, food, drink, work—undergo certain strange and as yet inexplicable isomeric variations, and thereby become at times various instruments having functions or duties changing from the normal, to the abnormal, and back again to the normal. Might it not be that the frequent absence of pathological distinctions after various forms of disease may be attributable to undiscovered and undiscoverable changes of isomeric kind? In varied forms of insanity, for instance, could it be that isomeric structural changes took place in nervous matter? Could sleep be the result of isomerical modification? Could there be such a thing as isomeric periodicity? *

Leaving the study of isomeric conditions changeable or interchangeable in the body itself, this question of isomerism in the chemical compounds that were under investigation remained and remains. The inference is that in one substance the elements are compounded or combined in one form, in another in a different form. But whatever may be the explanation, we have to recognise isomerism as an interference with the hypothesis of chemical construction as a guide to physiological action on living matter.

* Bearing on these points the late Professor Laycock, of Edinburgh, on hearing of them wrote begging me to let nothing stand in my way to hinder the continued study of the subject. He was good enough to say that he believed they would lead to an inauguration in medicine, especially as to the morbid states connected with insanity in its seemingly numerous but after all few typical or truly distinct forms. Unfortunately, a thousand other different and more immediately practical concerns of life have stood in the way of the pursuit of this friendly, and I know sincere, advice, and I fear the day is too far spent now.

